# Proteomics and phosphoproteomics in precision medicine: applications and challenges

**DOI:** 10.1093/bib/bbx141

**Published:** 2017-10-25

**Authors:** Girolamo Giudice, Evangelia Petsalaki

**Affiliations:** European Molecular Biology Laboratory European Bioinformatics Institute

**Keywords:** proteomics, phosphoproteomics, data integration, precision medicine

## Abstract

Recent advances in proteomics allow the accurate measurement of abundances for thousands of proteins and phosphoproteins from multiple samples in parallel. Therefore, for the first time, we have the opportunity to measure the proteomic profiles of thousands of patient samples or disease model cell lines in a systematic way, to identify the precise underlying molecular mechanism and discover personalized biomarkers, networks and treatments. Here, we review examples of successful use of proteomics and phosphoproteomics data sets in as well as their integration other omics data sets with the aim of precision medicine. We will discuss the bioinformatics challenges posed by the generation, analysis and integration of such large data sets and present potential reasons why proteomics profiling and biomarkers are not currently widely used in the clinical setting. We will finally discuss ways to contribute to the better use of proteomics data in precision medicine and the clinical setting.

## Introduction

Precision medicine refers to the use of diagnostic, therapeutic and monitoring strategies for individual patients based on their molecular profiles [1]. While there has been one promising example of monitoring molecular data from a single individual for a long term to assess their health and disease status [2], in practice, the focus of the community lies mainly in the stratification of diseases into subtypes, based on molecular biomarkers or signatures, i.e. in the molecular taxonomy of disease [3]. The aim is to use these signatures to assign patients to specific disease subgroups and administer the most effective therapy for them. For example, patients with certain variants of TPMT, a thiopurine methyltransferase, are known to exhibit severe toxicity to the most common leukemia chemotherapy drug, thiopurine [4]. The dosage of the drug for their treatment is thus currently adjusted, based on TPMT variant screening, to avoid the toxicity and treat leukemia effectively [5]. Extensive molecular characterization of gene expression signatures in breast cancer [6–8] has allowed the development of multigenes assays that are currently undergoing clinical trials for routine use in the clinic to guide patient treatment and monitoring [9].

**Figure 1:**
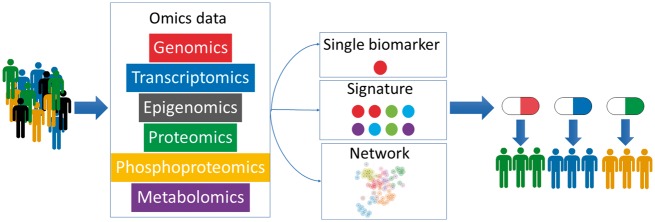
Example workflow for precision medicine. Multi-omics data are initially collected from patients and integrated to create their individual molecular profiles. These profiles are then matched to previously defined disease profiles that can guide the selection of treatment. This is achieved either through a match to known biomarkers, omics signatures or network/pathway signatures. The appropriate drug is then selected based on this match, to improve the chance of successful treatment and reduce the probability of side effects.

Most efforts to molecularly characterize diseases use genomic-based methodologies to identify genetic variants, including copy number variations [10] and differential gene expression [6] associated with specific disease subtypes [11] (Figure 1). While significant progress has been made in stratifying patients and diseases, there has been limited success in using this information in the clinic. In a recent meta-analysis study of a Phase 1 trial for treating refractory malignant neoplasms, they found that, while the response rate using the ‘precision’ biomarker was significantly higher than in its absence, the median response rate was still only ∼30% [12]. Systems biology [13] has shown that focusing only on the genomic and transcriptomic layers of cell function regulation leaves us blind to other important regulators of cell phenotypes and outcomes. For example, metabolomics data provide information regarding the metabolism and energy balance regulation of the cell, and epigenomics can reflect the regulation of the gene expression and the effect of environmental factors on the cell. The use of these data sets in precision medicine has been reviewed elsewhere [14, 15].

It is well known that changes in gene expression do not always reflect changes in protein abundance [16–18]. Proteins are the major effectors of cell functions through changes in their posttranslational modifications (PTMs) and abundance, reflected also on changes in their interactome with effects on cell phenotypes. It is therefore critical to also consider proteomics, phosphoproteomics and other PTM-‘omics’ data sets in our studies to understand disease development and subtypes, as they can better capture the functional state and dynamic properties of a cell. However, these data sets have not been extensively used in the precision medicine field because of the time required to run samples, complexity and dynamic range of proteomics samples, lack of reproducibility among laboratories, differences between quantification methods and other confounding factors [19, 20].

Recently, technological developments in instrumentation, sample preparation and data analysis [20–23] and initiatives to develop standards for the generation and evaluation of these data [24–30] have resulted in the availability of high-quality, reproducible and comprehensive proteomics and phosphoproteomics data sets and protocols to generate such data. For example, Sharma and colleagues [31] were able to detect 50 000 phosphopeptides in a single human cancer cell line, and scientists can routinely and accurately measure thousands of peptides within short time frames: Hebert *et al.* [32] were able to measure the entire yeast proteome comprising peptides from ∼3980 proteins in just over 1 h. Hundreds of targeted and global proteomics data sets are also collected by the CPTAC (Clinical Proteomic Tumor Analysis Consortium) to contribute to the study of cancer [33]. Therefore, the bioinformatics community must currently address the challenge of taking advantage of this new layer of information and integrating it with other valuable omics layers to study the mechanism of human disease and translate it into actionable insight in the clinic. Targeted proteomics methods such as SRM/MRM (Selected/Multiple Reaction Monitoring; [34]) and data-independent acquisition methods such as SWATH-MS (Sequential Windowed Acquisition of All Theoretical Fragment Ion Mass Spectra) also allow significant reduction in variability during data acquisition and improved data set quality [35]. For details on the technological advances that have allowed this revolution in proteomics and PTM-omics data acquisition, we redirect the reader to numerous existing publications [21, 22, 36–38]. Recent reviews have discussed proteomics and phosphoproteomics in the context of precision medicine [39, 40]. In this review, we will present an overview of bioinformatics approaches used to analyze these data individually as well as integrated with other omics data sets and will discuss challenges that should be tackled to gain insight into disease mechanisms and advance the field of precision medicine.

## Proteomics-derived precision biomarkers and signatures

A major application of proteomics is for the identification of biomarkers for disease. Biomarkers can be divided in (i) diagnostic to identify a given type of disease (ii) prognostic to measure the disease status and (iii) predictive to measure a response to a treatment [41]. Ideally, a biomarker should distinguish the disease unambiguously and should be detected in an accessible body fluid such as plasma, blood, serum urine, saliva or cerebrospinal fluids [42]. For example, the prostate-specific antigen (PSA) is one of the most famous noninvasive screening biomarkers and is used to detect prostate cancer [43]. However, a high concentration of *PSA* in the blood is also associated with benign prostatic hyperplasia and prostatitis [44–46]. Thus, even though *PSA* provides sufficient sensitivity, it fails in the discrimination between prostate cancer and other prostate pathologies because of its poor specificity [47]. In recent years, to improve biomarker sensitivity and specificity, researchers have turned to a combination of biomarkers, i.e. a disease signature, instead of pursuing an ideal biomarker [48].

Using proteomics characterization of samples from different stages of luminal-type breast cancer progression, Pozniak *et al.* [49] identified differences in components of protein homeostasis and metabolic regulation that can differentiate healthy, from primary or lymph node-metastasized tumor tissues, and lymph node-positive and negative breast cancers. Proteomics-based subtyping of colon and rectal cancer patients by the CPTAC was also more fine-grained than that based on transcriptomics data leading to better prediction of patient prognosis [50]. Combining protein with phosphoprotein abundance measurements using reverse phase protein arrays has also been used, e.g. for the prediction of ovarian cancer recurrence [51]. Numerous studies have showcased the value of phosphoproteomics data in providing mechanistic information underlying the disease mechanism [52–54]. For example, phosphoproteomics data have been used to discover the mechanism of resistance of melanoma cells to *BRAF* inhibitors [52] and of glioblastoma to *mTOR* (mechanistic target of rapamycin) inhibitors, leading to the discovery of a novel combination therapy for the latter [53]. Casado and collegues [55] used phosphoproteomics data on hematological cancer cell lines to assign them to specific tumor types and potential treatments. They also studied acute myeloid leukemia primary cells to identify the differential activation of kinases in cells that presented different drug resistance profiles [56]. Excitingly, cell-specific phosphoproteomics has also been used to study bidirectional signaling between endothelial cells and tumor cells to understand metastatic mechanisms of tumor cells [54]. Recently, phosphoproteomics data were used to create mechanistic models of colorectal cancer cell line-specific drug resistance, suggesting that this could be a viable option also for patients [57]. It is therefore clear that the proteomics and phosphoproteomics layer of omics information can provide valuable insight in our quest towards precision medicine.

Extracting relevant and reliable features (proteins) from high-throughput proteomics data is the main challenge for the biomarkers identification process. One approach is to use those proteins that are differentially expressed between normal and disease state [58–62]. More sophisticated methods such as machine learning and network-based approaches are also used. Machine learning methods such as support vector machine [63, 64] (SVM), neural networks [65–69], decision tree [67], random forest [70, 71] and genetic algorithms [72] have been successfully applied to proteomics data to identify biomarkers for several cancer types, heart failure and other conditions. Ahn *et al.* [73] constructed a 29-plex array platform comprising 29 potential biomarkers associated with gastric adenocarcinoma. A total of 13 candidate biomarkers were selected by random forest feature selection algorithm. Random forest and SVM were used to classify individuals as patients with gastric adenocarcinoma or controls. The algorithms tested on an independent blinded set of 95 gastric adenocarcinoma sera and 51 controls reached a mean accuracy of 89.2 and 85.6%, respectively. Random forest generally outperformed SVM, regardless of stage or tumor size; however, the SVM algorithm performed well for diagnosing small tumors. Rogers *et al.* [66] trained a neural network on either presence/absence of peaks or peak intensity values in a cohort of patients affected by renal cell carcinoma. Their model reaches sensitivity and specificity values of 98.3–100%. However, in an independent validation cohort of 80 cases, the performances were significantly weaker (sensitivities and specificities ranged from 41.0 to 76.6%). This highlights the frequent tendency of machine learning approaches to overfit their functions to noise inherent to the data set rather than the signal. Appropriate consideration regarding the complexity of the model and control data sets should thus always be used to avoid this issue when using such approaches.

High-throughput proteomics data sets are characterized by a high number of variables/features compared with the total number of samples available. Hence, the input space includes many irrelevant or noisy features, which, coupled with the wide heterogeneity commonly found in biological samples, make it difficult to identify the truly important biomarkers. To tackle this problem, dimensionality reduction methods [74], such as PAM (Prediction Analysis for Microarrays) [75], SVM-RFE (Support Vector Machine-Recursive Feature Elimination) [48], SAM (Significance Analysis of Microarrays) [76], are used, in combination with machine learning methods, to reduce the noise in the data sets. This is achieved by discarding irrelevant features and enhances the generalization and the prediction performance. For reviews of feature selection algorithms, we redirect the reader elsewhere [77–79].

The lack of reproducibility across different data sets, technical issues such as the overfitting problem in machine learning approaches and the intrinsic complexity of human diseases often prevent promising biomarkers from reaching clinical application [80]. A promising idea to improve the reproducibility and the interpretation of the results is to incorporate prior biological knowledge and different high-throughput data sets to facilitate our understanding of biological processes at a mechanistic level.

## From lists to integrated networks

**Figure 2:**
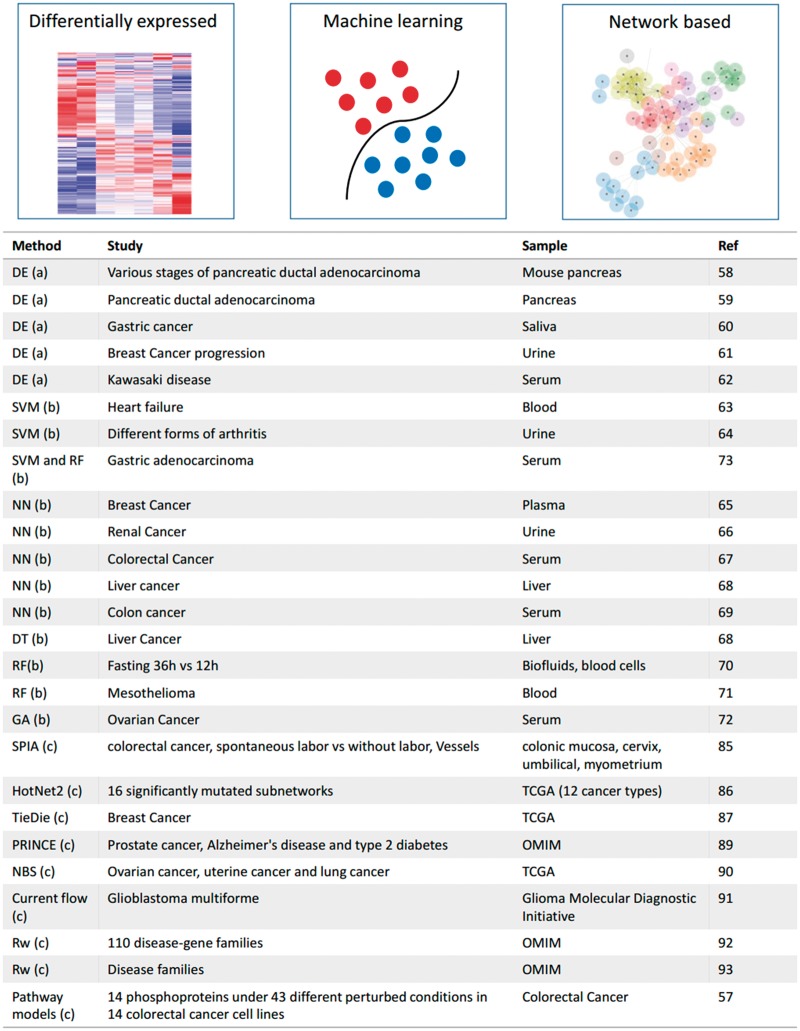
Different methods used in biomarker discovery. (**A**) Differentially expressed method, (**B**) machine learning method, (**C**) network-based method DE, differentially expressed; NN, neural network; RF, random forest; DT, decision tree; GA, genetic algorithm; NBS, network-based stratification; RW, random walk.

Uncovering the individual mechanisms of disease development and progression in different patients will be key to designing accurate precision therapy strategies. As a first step in that direction, omics data analysis approaches typically attempt to identify affected biological processes and functions [49] by using Gene Ontology [81] or pathway (or other features) enrichment analyses [82] on the differentially regulated entities of each data set (e.g. genes, proteins or phosphopeptides). These differentially regulated entities can also be mapped onto existing interaction networks or pathway maps to provide a better picture of the cell processes affected in a specific sample. For example in the tumor endothelial bidirectional signaling study mentioned above [54], the authors mapped the affected phosphopeptides onto KEGG pathway maps [83], to understand the pathways involved in the transendothelial metastasis of tumors. More recently, a collection of methods, mostly developed for and applied to genomics and transcriptomics data sets, has been developed that take into consideration also the protein interaction network and pathway structure to identify patient-specific disease-perturbed pathways [84]. The SPIA algorithm (Signalling Pathway Impact Analysis) combines information on the differential expression of genes with their influence in a pathway based on their placement in a pathway topology [85]. HotNet2 [86] and Tied Diffusion Through Interacting Events (TieDIE; [87]) use slightly varied diffusion-based approaches that include a form of random walk and weighting according to the connection strength and network topology to propagate the effect of the perturbation in a given network [88]. There are many other methods available (the most widely used are reviewed here [84]) using, for example, network propagation [89] and clustering [90], current flow through the network [91], random walk [92, 93], pathway models [57] or other approaches for identifying perturbed functional modules or pathways in a network and using these as signatures to stratify patients or differentiate cancer model cell lines (Figure 2).

The concepts and methodologies can also be applicable to proteomics data sets; however, there are some issues that should be considered both when using these methods for transcriptomics/genomics data and when attempting to apply them to proteomics and phosphoproteomics data sets. Specifically, most of them tend to use existing interactome data and annotated pathway data, which are currently incomplete and biased toward highly expressed proteins [94–96]. This issue is further exacerbated, when trying to apply them to proteomics data sets, by the fact that these also inherently contain this bias. Moreover, our knowledge of tissue-specific interactions and their rewiring in different cellular states or conditions is currently limited [97, 98]. Such rewiring also occurs in disease and may vary across patients, and therefore, the use of generic networks and pathways for precision medicine applications may not be ideal. Finally, another issue to consider when applying such methods to proteomics and phosphoproteomics data sets is that they tend to have a much smaller coverage of the entire proteome than other respective omics data sets, depending on the instrument or technology used and the dynamic range of the abundances in the sample [99, 100]. It would therefore be useful to develop computational approaches that are tailored specifically to proteomics and phosphoproteomics data sets to account for these associated data characteristics.

In the past few years, there have been a number of such methods developed. They mainly focus on accurately estimating the activity of diverse kinases in the systems under study to highlight the context-specific signaling networks that are active in each context. The most widely used method is the kinase–substrate enrichment analysis method [56], which calculates the kinases activity based on the differential abundance of their known substrates. Other methods include IKAP (inference of kinase activities from phosphoproteomics; [101]), which uses a machine learning approach, KARP (kinase activity ranking using phoshphoproteomics data; [102]), which calculates the relative phosphorylation of a kinase‘s substrates versus the total phosphorylation in the data and KinasePA (Perturbation analysis; [103]) and CLUE (CLUster Evaluation; [104]), which require perturbation or time series data. A few different approaches have been extensively benchmarked by Hernandez-Armenta and colleagues [105].

As proteomics and phosphoprteomics data sets provide a direct picture of the cell’s functional state, inclusion of prior knowledge in these methods, such as motifs or interaction interfaces, known enzyme–substrate relationships and effects of mutations on protein structure and function, can also help better understand the effect of perturbations on the functional network.

### Data integration approaches

Despite the wealth of information that proteomics and phosphoproteomics data can provide, it still represents only one layer of cell function and regulation. Thus, to truly understand cell function in-depth, it is critical to consider as many as possible layers of cell function regulation [13]. This is especially true in the context of precision medicine where different layers of cell regulation may be important for each patient, and additional clinical information must also be included in the analysis. Therefore, one major challenge that our community is currently trying to solve is that of effective data integration of the less mature proteomics and phosphoproteomics layers of information with other omics data sets that have been more extensively studied and integrated in recent years.

There is currently no standard or optimal approach to data integration, and several methods have been developed (for reviews, see [106–108]). Here, we will focus on the main approaches used thus far to integrate proteomics data sets with other omics or clinical data. Depending on the data sets they integrate, methods can be divided into homogenous, where the data sets contain the same type of data but from different sources, and heterogeneous, where multiple data sets with different data types are integrated. These methods can either integrate the layers of information in a step-wise fashion or in a single step to generate an integrated model of the system under study.

For example Drake *et al.* [109], used a step-wise approach to integrate genomic, transcriptomic and phosphoproteomics data to identify patient-specific networks that are affected in prostate cancer and suggest potential precision treatments for these patients. Specifically they first used the data sets to broadly identify the pathways, transcription factors and kinases that are likely active in their samples and then applied their diffusion-based algorithm, TieDie [87], to pinpoint the different functional modules and pathways that are affected in the different patients. In this study, they also showed that the integration of phosphoproteomics was able to uncover pathways that would have otherwise been missed underlining the importance of including this level of information in precision medicine approaches. By applying this pipeline on three different prostate cell lines as validation, they were able to support their results either through evaluation of their predicted drug sensitivity or through gene essentiality studies.

Rudolph and colleagues [110] integrate protein interaction networks with phosphoproteomics data and evolutionary conservation to define signaling functionalities for proteins in a data set and delineate the active signaling pathways in a given phosphoproteomics data set. A recent systematic search for algorithms to reconstruct signaling pathways from phosphoproteomics [111] has shown that integration with prior knowledge yields the best results.

The most promising methods that integrate data sets in a single step include principle component analysis (PCA) [112] (or factor analysis)-based and nonnegative matrix factorization (NMF)-based [113, 114] approaches, as they are able to integrate diverse and large data sets and perform effective dimensionality reduction to allow easy downstream machine learning [115] or network-based [113] analyses and creation of models that represent the system under study.

The major issue with PCA-based approaches is the difficulty in interpretation of the biological mechanism underlying the different factor associations. Therefore, different supervised [116] or unsupervised [117] approaches can be used to choose the appropriate factors and help the results interpretation. These can include implementation of linear discriminant analysis [116], Bayesian classifiers [118], SVMs [119] and *K*-nearest neighbor [120] approaches after the PCA analysis. Liu *et al.* [118] integrated microRNA, mRNA and proteomics data into a joint matrix. They then used factor analysis and linear discriminant analysis to extract the molecular mechanism of cancer in different cell lines. The integrated approach identified clinically relevant markers and outperformed the analyses performed on the separate data sets.

While matrix factorization methods such as NMF and variations have been routinely applied to genomics and transcriptomics data [113, 121, 122], they have only recently been applied to proteomics data sets. For example, Yuan *et al.* [123] used pairwise NMF between omics data sets and clinical data to study the utility of using these omics data integration approaches in the clinic. In the subgroups, which they identified by combining proteomics and clinical data, they were able to identify—among other biomarkers and activated pathways—an additional patient subgroup that might also benefit from *MEK* (Mitogen-activated protein kinase kinase) targeting therapies.

A great advantage of matrix factorization approaches for proteomics and phosphoproteomics data sets is that they can also be used to impute missing data points [124]. This can be valuable for these data sets, as they inherently do not provide comprehensive measurements of all the components that might be present in other omics data types such as transcriptomic or genomic data sets. Other approaches for data imputation that can be applied in proteomics and phosphoproteomics data use nonlinear optimization approaches [125, 126].

Another integration approach that has been applied to the proteomics data is based on a multiple extension co-inertia analysis to identify the relationships among different omics data sets. Meng *et al.* [127], for example, integrated the transcriptome and proteome profiles of cells in the NCI-60 cancer cells. Using the integrated model, they found that the extravasation signaling pathway plays a fundamental role in leukemia; the same pathway was not identified in the single data set analyses.

Other than the missing data points that were discussed above, one of the major challenges for integrating proteomics and phosphoproteomics with other omics data sets is the inconsistent annotation and reporting of such data sets and analysis pipelines. This, in combination with the dynamic nature of the proteome and phosphoproteome, can result in the introduction of noise to the integrative models used to study a disease or a patient. As unified data collection and standardization processes are being developed for use of these data in the clinic, consistent methods to record the associated meta-data for this information that can be used in conjunction with existing methods for genomic and other omic data sets need to also be developed.

In recent years, there have been bioinformatics platforms and methods developed to reduce the variability from the data acquisition and analysis processes. Examples for this are the ProHits [128] and OpenMS [129]. ProHits is a software platform that is used mainly for interaction proteomics and provides a variety of options for data management and analysis that are systematically tracked to ensure the downstream reproducibility of the analysis pipelines. OpenMS is an open-source suite of analysis software for mass spectrometry data allowing the implementation of different pipelines and analyses procedures in a transparent and scalable way. These kinds of platforms ensure the reproducibility of the analyses pipelines. Methods for ensuring reproducibility during data acquisition are also important. For example, the TRIC (Transfer of Identification Confidence; [130]) algorithm, developed for SWATH-MS-targeted proteomics, uses a clever alignment approach to reduce the variability in peak picking and quantification across mass spectrometry runs. Other similar software has been previously compared by Navarro *et al.* [131].

The inherent variability of proteomics and phosphoproteomics data sets can also be a confounding factor in data integration efforts. It has been shown in single-cell studies that the noise and sample variability significantly decrease when a specific cell response is activated compared with a static state, because of regulatory coordination [132]. Therefore, acquiring nonstatic data points, where possible, will reduce data variability and increase the signal to noise ratio. Additionally, single-cell technologies, providing single-cell measurements of protein or phosphoprotein abundance, have the potential to mitigate the data variability issue and improve the use of these data for understanding disease development.

### From networks to mechanistic models

For use of proteomics and phosphoproteomics in the clinic, it is important to provide mechanistic information for a disease beyond the pathways and functional modules that have been affected. Halasz *et al.* [133] used phosphoproteomics data sets and a probabilistic framework to create a mechanistic and executable model of the rewiring that occurs in signal transduction pathways in cancer cells. They were able to identify a cell line-specific feedback loop for inhibition of IRS1 by p70S6K in colorectal cell lines and to perform stimulations to identify ways to increase their sensitivity to TRIC (*TCP-1* ring complex) inhibitors. Eduati *et al.* [57] used dynamic logic models and phosphoproteomics data to study the colorectal cell line-specific mechanism of drug resistance and a identified novel drug combination that can be used to overcome it.

Such models can be invaluable in the clinic to not only understand the mechanism of disease but also to simulate and predict the outcome of a treatment on specific patient groups or even individuals, depending on the available models.

## Challenges for clinical application

While the proteomics and phosphoproteomics layers of functional regulation provide valuable insight into disease development and mechanism, there are still some challenges that need to be tackled before they can be readily applied for stratification of patients, even if data quality and bioinformatics challenges discussed above are tackled.

One of the major challenges is that most current ‘omics’ data analyses provide results that are not readily interpretable or actionable. For example, while identifying that a handful of pathways are affected in a specific patient subgroup may suggest the administration of specific kinase inhibitors as therapy, it does not necessarily uncover the full mechanism of a disease. There have been successful examples, such as the work of Zeevi and colleagues [134] that used omics data, clinical data and machine learning to devise an actionable change in personalized nutrition to regulate post-meal glucose levels, without an in-depth understanding of the mechanism at play. However, in most situations, lack of mechanistic information regarding a disease’s development, makes it difficult to identify the causal targets for therapy at a reliability level that is appropriate for precision therapies in the clinic. As new methods for proteomics data analysis develop, our community needs to take this into consideration: rather than providing ‘big picture’ representations of affected cell processes in a disease, there is a need for producing reliable ranked targets or biomarkers by probability of being effective [135–137] or ranked testable hypotheses to help decide on one, alongside an easy-to-interpret explanation for their selection. This requires an in-depth understanding of cell processes and their interactions.

Recent years have seen the collaborations between computational biologists and clinicians or basic-science biologists dramatically increase, because of the advent of large-scale data sets and systems biology. The importance, however, of understanding basic biological processes in-depth to be able to understand disease mechanisms underlines the need for increased collaboration also between clinicians and basic research scientists. Interdisciplinary collaborations, including clinical data to take snapshots of the disease ‘omics’ profile, and iterations of computational analysis and basic biology for in-depth mechanistic studies of relevant cell processes, can lead to a detailed understanding and models of disease development, thus helping better stratify patients according to their disease subtype mechanism and design more knowledge-based treatments. Proteomics and phosphoproteomics data sets, as described above, can provide mechanistic insight into cell processes and are therefore ideal for inclusion in such studies to provide testable mechanistic hypotheses. Of course, the major disadvantage of such three-level approaches is that it takes time to perform in-depth studies of cell processes; however, as our knowledgebase of cell processes, their cross-talk and their role in different diseases increases, this will prove to be a worthwhile investment in the long run and might be the only way to truly achieve the goal of precision medicine across multiple diseases.

An additional challenge is presented when associating identified affected cell processes with specific disease phenotypes or clinical data. Currently, most studies use patient survival data as the patient phenotype and associate omics signatures with remission or survival rates [138]. More detailed and standardized phenotyping of patients can provide a better understanding of the causal cell processes of a disease and can improve diagnosis and tracking both of its progression and the effects of treatment and other issues that might affect a patient’s quality of life [138]. As more omics data from patients are being generated, standardized protocols for systematically recording the phenotype of the relevant cells—if possible—and wider availability of in-depth patient clinical characteristics to data scientists beyond survival rate will also provide a significant contribution toward our community‘s goal of precision medicine. Ethical considerations to ensure patient anonymity and privacy need to also be taken into account in the development of these protocols as well as in the process of data sharing [140, 141].

The standardization of analysis pipelines and representation of results also present an issue for the routine application of proteomics protocols in the clinic. Whether the outcome of patient data analysis is the identification of a biomarker or a disease signature, robust quality control and analysis tools needs to be readily available to clinicians as well as accurate protocols for sample acquisition and results interpretation. This is critical to provide reproducible, high-quality precision care for patients across different hospitals and treatment centers. Proteomics and phosphoproteomics-specific data analysis pipelines have only recently started to be systematically developed and included in precision medicine studies [109]. Therefore, as the field matures, we expect to see significant progress in their standardized use across laboratories, institutes and eventually in the clinics.

## Future directions

Proteomics and phosphoproteomics have recently emerged as a new layer of patient omics information in the field of precision medicine. Technological advancements and community efforts to standardize protocols and achieve robust and reproducible results [24–30] have contributed greatly to the utility of this data type in large-scale studies of disease and patient stratification. Their major strength lies in the fact that they give a picture of the actual workforce of the cell and are thus highly suited for studying the mechanism of disease development and progression. Other than the data reproducibility issue that the community is now efficiently tackling, one of the main challenges from a bioinformatics perspective that still prevents the wide-spread use of proteomics and phosphoproteomics data is the need for effective, data type-specific methods to extract the valuable knowledge it encodes and to integrate it efficiently with other large-scale data sets and prior knowledge. There are significant efforts made in this direction, and as the field matures, and more PTMs are also included, we expect it to provide great insight into the development of disease and help improve stratification of patients and design of precision approaches to their treatment and monitoring. Additionally, proteomics and phosphoproteomics data, like transcriptomics, encode highly dynamic information. Therefore, to accurately highlight differences in disease mechanisms and functional networks, and to reduce data variation, it is optimal to collect data sets on stimulation or perturbation rather than in a static state. This is currently impractical in a clinical setting, where we rely on a single sample from a usually untreated patient, but it could prove useful when performing, for example, window of opportunity trials where novel drugs are tested on patients before the standard treatment to evaluate their effect on untreated individuals [142, 143].

Currently, the bulk of population-level omics data is being collected to study cancer for precision oncology applications. Clearly, for precision medicine to become widely applicable more focus should be placed on characterizing also other diseases and their subtypes. These cancer studies, nevertheless, provide a unique learning opportunity for our community: we can use this rich data set to define what is the best way to maximize the orthogonal information we acquire from all these different omics layers, to estimate how many data sets are sufficient for characterizing a disease and potentially to identify the minimal components that one needs to measure in a cell to get the global signaling, gene regulation and metabolic status from a sample. From a proteomics perspective, such information can dramatically reduce the cost and variability of a study, making it even more applicable for clinical applications, for example through an educated design of targeted proteomics or phosphoproteomics approaches.

Of the drugs that are tested in clinical trials only 1 in 10 successfully go to the market [144]. This presents a huge financial burden for the pharmaceutical companies and the public. Bioinformatics approaches that effectively integrate omics data with in-depth clinical data can help guide many aspects of clinical trials to improve the chances of their success (for a recent review, see [145]): analysis of patients’ omics data can help to guide the selection of targets and associated drugs and the appropriate group to which a drug can be administered with improved chance of success. Bioinformatics data storage and automated analysis pipelines can also make this knowledge available to future studies. At later stages, side effects or outcomes of the trial can be associated with specific molecular signatures in the patients to understand their mechanisms and design approaches to circumvent them. Indeed, these methods are already in use, and there are already guidelines in place to guide the design of clinical trials using omics data sets [146]. Thus, as an increased amount of clinical records and associated omics data sets become available to scientists, bioinformatics approaches will play an important role in guiding clinical trials with an increased success rate.

In an ideal precision medicine scenario, we would be able to create a widely used and robust clinical tool that can guide doctors with respect to the data required from a patient to provide his subdisease mechanism and guide the choice of therapy and monitoring. While we are several decades away from such a tool, and indeed from widespread use of any precision medicine approaches at all, it is nevertheless becoming increasingly clear that understanding at the molecular level and creating dynamic mechanistic models of cell functions during disease development and progression are critical for the success of precision medicine.

Precision medicine for all is still a long-term goal for our community. However, the field is rapidly progressing, and it certainly does not seem as far-fetched as it did 10 years ago. Even not taking into consideration the improvement in global quality of life, studies have demonstrated the cost–benefit of applying such approaches in the clinic [147].

Programs such as the St Jude Children’s Research Hospital Pharmacogenomics of Anticancer Agents Research 4Kids (PG4Kids) program [148] and the Icahn School of Medicine at Mount Sinai Clinical Implementation of Personalized Medicine through Electronic Health Records and Genomics-Pharmacogenomics (CLIPMERGE PGx) program [149] can provide valuable knowledge regarding the practical prerequisites for real life precision medicine implementation. Additionally, exciting developments in preclinical studies include the use of patient-derived xenograph mouse models of disease (e.g. at the Jackson Laboratory), for testing precision therapies. We expect current and future advances in proteomics and phosphoproteomics data collection and analysis to greatly improve our understanding of disease development and progression also contributing to improved implementation of precision medicine in real world applications.

## Notes

Key Points

- Precision medicine aims to tailor diagnostic, therapeutic and monitoring approaches to specific patient subgroups.
- Proteomics and phosphoproteomics data sets can provide mechanistic insight into disease development and are thus valuable for precision medicine approaches.
- Major challenges presented by these data include the lack of data robustness and standardization as well as the limited proteome and phosphoproteome coverage.
- Methods that are developed specifically for these data types as well as their effective integration with other data sets can mitigate the issues.
